# Growing challenges in nursing education: prevalence and mediating role of computer vision syndrome in the relationship between visual fatigue and sleep disturbance among nursing students

**DOI:** 10.1186/s12912-025-04167-6

**Published:** 2025-12-25

**Authors:** Mohammed Elsayed Zaky, Shimmaa Mohamed Elsayed, Nourah Alsadaan, Shaimaa Ahmed Awad Ali, Shimaa Magdy Farghaly

**Affiliations:** 1 Faculty of Nursing Cairo University, Medical Surgical Nursing Department, Cairo, Egypt; 2 Critical Care and Emergency Nursing Department, Faculty of Nursing Damanhour University, Damanhour, Egypt; 3 Present Address: College of Nursing, Jouf University, Department of Nursing Administration and Education, Sakaka, Saudi Arabia; 4 Present Address: Critical Care and Emergency Nursing Department, Faculty of Nursing, Mansoura University, Mansoura, Egypt; 5 Department of Medical Surgical Nursing, College of Nursing, Jouf University, Sakaka, Saudi Arabia

**Keywords:** Nursing Education, Vision disorders, Nursing students, Ergonomics

## Abstract

**Background:**

The digital transformation of nursing education has heightened screen exposure, raising concerns about Computer Vision Syndrome (CVS), visual fatigue, and sleep disturbances. However, the mediating role of CVS in linking visual fatigue to sleep quality remains underexplored in this population.

**Aim:**

To investigate the prevalence of CVS, visual fatigue, and sleep disturbances, and to explore CVS as a mediator between visual fatigue and sleep quality among nursing students.

**Methods:**

A cross-sectional study involving 214 nursing students was conducted. Data was collected using validated tools: the Computer Vision Syndrome Questionnaire, Visual Fatigue Scale, and Sleep Quality Scale. Descriptive statistics, Pearson’s correlation, linear regression, and path analysis were employed for analysis.

**Results:**

CVS prevalence was 50.9%, with moderate visual fatigue (mean = 18.06) and sleep disturbances (mean = 33.66). CVS and visual fatigue were strongly correlated (r = 0.675, p < 0.001) and positively associated with sleep disturbances (r = 0.455 and r = 0.436, respectively, p < 0.001). Path analysis indicated that CVS directly affected sleep quality (β = 0.296, B = 0.816, p < 0.001) and visual fatigue directly affected CVS (β = 0.675, B = 0.315, p < 0.001). The indirect effect of visual fatigue on sleep quality via CVS was also significant (β = 0.200, B = 0.257, p < 0.001).

**Conclusion:**

Computer Vision Syndrome (CVS) and visual fatigue significantly affect sleep quality among nursing students, with CVS partially mediating this relationship. These findings highlight the workplace health implications of digital learning in nursing education, emphasizing the need for interventions to reduce digital eye strain and improve sleep quality in this population.

## Significance

Implications for Profession and/or Patient Care

Impact

Reporting Method

Patient or Public Contribution

What Does This Paper Contribute to the Wider Global Clinical Community?

What is Already Known

Implications for Practice/Policy

This study enhances the understanding of how Computer Vision Syndrome (CVS) and visual fatigue influence sleep disturbances among nursing students, highlighting the mediating role of CVS in this relationship. It underscores the importance of addressing digital eye strain and visual fatigue to improve sleep quality and overall well-being in nursing education.

The findings suggest that nursing educators and healthcare policymakers should prioritize interventions to reduce CVS and visual fatigue. Implementing preventive measures could mitigate the negative effects of prolonged screen use on sleep quality, ultimately enhancing academic performance and patient care.

(STROBE) checklist was used for reporting current study.

No patient or public contribution was involved in this study.

This study highlights the mediating role of Computer Vision Syndrome (CVS) in the relationship between visual fatigue and sleep disturbances among nursing students. It emphasizes the need for interventions to address digital eye strain and visual fatigue, which are critical for improving sleep quality and overall well-being in nursing education. The findings are relevant to the global clinical community, as they underscore the importance of promoting digital ergonomics and screen time management, particularly in healthcare education and practice settings where digital device use is prevalent.

•Prolonged screen use is associated with Computer Vision Syndrome (CVS), visual fatigue, and sleep disturbances.

•Nursing students, due to their reliance on digital devices for academic and clinical tasks, are particularly vulnerable to these issues.

•The mechanisms linking CVS and visual fatigue to sleep disturbances remain underexplored, especially in the context of nursing education.

•Nursing educators and healthcare policymakers should prioritize interventions to reduce CVS and visual fatigue, such as promoting digital ergonomics, screen time management, and awareness campaigns.

•Implementing preventive measures, such as regular screen breaks, proper lighting, and blue light filters, could mitigate the negative effects of prolonged screen use on sleep quality.

•These findings are particularly relevant for nursing education programs, where digital device use is integral to learning and clinical training.

By addressing these issues, this study contributes to the global clinical community by providing actionable insights to improve the health and well-being of nursing students, who are essential to the future of healthcare delivery.

## Background

The digital transformation of nursing education has accelerated in recent years, driven by the increasing integration of technology into academic curricula and the widespread adoption of online learning platforms [1]. Virtual simulations, electronic textbooks, and video-based instruction have become fundamental components of modern nursing pedagogy, enhancing accessibility and flexibility for students [2]. However, this shift has also resulted in significantly increased screen exposure, with nursing students spending prolonged hours engaging with digital devices for lectures, assignments, and clinical simulations [3].

While digital learning offers numerous benefits, excessive screen time has been associated with various health concerns, including ocular discomfort, mental fatigue, and sleep disturbances [4]. Among these, computer vision syndrome (CVS)a cluster of eye and vision-related problems induced by prolonged digital device use has emerged as a growing concern in nursing education [5]. CVS is characterized by symptoms such as eye strain, dryness, blurred vision, and headaches, often exacerbated by poor ergonomics, prolonged screen exposure, and reduced blinking rates [6]. Studies indicate that CVS is highly prevalent among students in health-related disciplines, where extensive screen use is necessary for academic success [7].

Nursing students, in particular, may be at heightened risk due to the combined demands of digital coursework and electronic health records training, leading to increased ocular fatigue and visual discomfort [8]. Notably, eye fatigue, a core symptom of CVS, has been linked to broader physiological effects, including impaired sleep quality and circadian rhythm disruption [4].

## Introduction

The relationship between eye fatigue and sleep disturbances is well-documented, with digital exposure contributing to delayed melatonin secretion, prolonged sleep onset latency, and reduced sleep efficiency [9]. However, the potential mediating role of CVS in this relationship remains underexplored, particularly among nursing students who rely extensively on digital tools for their education. Understanding this interplay is crucial in mitigating the negative consequences of prolonged screen exposure in nursing education.

The cyclical nature of eye fatigue, CVS, and sleep disturbances suggests a complex interdependence. Eye fatigue, resulting from prolonged visual strain, has been identified as a precursor to CVS, as it exacerbates ocular discomfort and disrupts normal visual processing [9]. In turn, CVS symptoms such as dry eyes, blurred vision, and headaches can increase physiological stress, further deteriorate sleep quality by inducing discomfort and prolonged cognitive arousal [10]. This interplay suggests that CVS may serve as a mediating factor, intensifying the impact of eye fatigue on sleep disturbances.

The digital transformation of nursing education has led to a marked increase in screen exposure among students, raising critical health concerns such as Computer Vision Syndrome (CVS), visual fatigue, and sleep disturbances. Recent evidence highlights the magnitude of these issues: a cross-sectional study among Peruvian nursing students reported a CVS prevalence of 87.6% [11], while studies in Saudi Arabia and Jordan indicated CVS symptom rates exceeding 70–90% among health sciences students [12]. In parallel, the prevalence of visual fatigue is often a precursor to or symptom of CVS has been reported in over 60% of university students exposed to prolonged digital device use [13].

Sleep disturbances are another growing concern in this demographic. A study found that between 40% and 70% of nursing students globally experience poor sleep quality, often associated with screen use, stress, and irregular schedules [14].

Given the increasing reliance on digital tools in nursing education, understanding how visual fatigue contributes to sleep disturbances through Computer Vision Syndrome (CVS) is essential for developing targeted interventions. While previous studies have examined the individual effects of visual fatigue and CVS on sleep quality, the potential mediating role of CVS in this relationship remains underexplored. This study proposes a mediation model in which CVS acts as a key intermediary between visual fatigue and sleep disturbances, providing a framework to identify points of intervention for improving digital health management among nursing students.

## Hypothesis

### H1:

Visual fatigue will be positively associated with the severity of Computer Vision Syndrome (CVS) symptoms among nursing students.

### H2:

Computer Vision Syndrome (CVS) will be positively associated with sleep disturbances among nursing students.

### H3:

Visual fatigue will be positively associated with the severity of sleep disturbance among nursing students.

### H4:

**Fig. 1:**
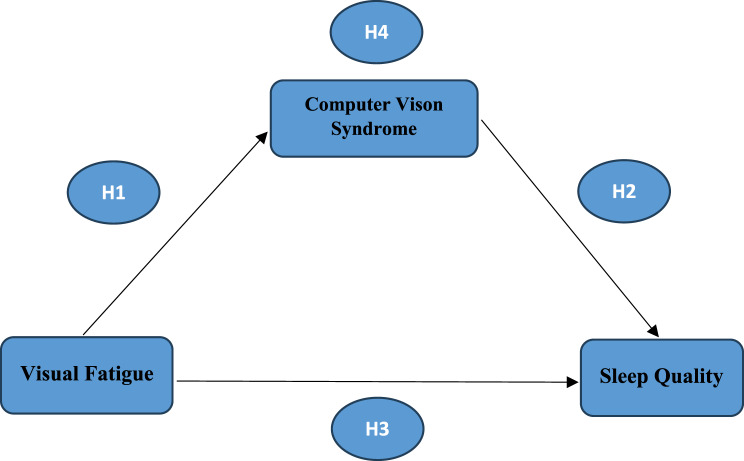
Proposed mediation Model with computer vision syndrome as the Mediator between visual fatigue and sleep quality

Computer Vision Syndrome will mediate the relationship between visual fatigue and sleep quality among nursing students (Fig. 1).

## Aim of the study

This study aimed to examine the prevalence of CVS, visual fatigue, and sleep disturbances, and to explore the mediating role of CVS in the relationship between visual fatigue and sleep quality among nursing students.

## Methodology

### Design

This study employed a quantitative, cross-sectional research design to explore the relationships between computer vision syndrome, visual fatigue, and sleep quality among nursing students. The study was reported in accordance with the Strengthening the Reporting of Observational Studies in Epidemiology (STROBE) checklist, which provides guidelines to improve the transparency, accuracy, and completeness of reporting observational studies, including cross-sectional research [15].

### Settings and participants

his cross-sectional study was conducted at a single institution, the Faculty of Nursing at Damanhour University, Egypt. A convenience sample of 214 undergraduate nursing students was recruited. The use of convenience sampling may introduce self-selection bias, potentially over-representing students who are more engaged with digital tools or more concerned about visual health. Participants were required to meet the following inclusion criteria [1]: currently enrolled in a nursing program [2], have used digital devices (such as computers, laptops, or smartphones) over the past 6 months. Exclusion criteria included [1]: Having neurological conditions (such as migraines or epilepsy) that could affect visual perception or sleep [3], use of medications that could influence vision or sleep quality, and [4] participants with a history of psychiatric disorders that might affect their sleep patterns or overall health.

### Sample size calculation

The sample size was estimated to be using an online a priori sample size calculator for structural equation modeling [16]. Parameters included a power level of 0.95, anticipated effect size of 0.3, significance level of 0.05, 3 latent variables, and 50 observed variables, resulting in a minimum required sample size of 184 participants. To account for potential dropouts, the target was increased by 30%, yielding a final target of 240 participants. From this pool, 214 valid responses were obtained, achieving an effective response rate of 89.2%. A post-hoc power analysis confirmed that the final sample size of 214 met the power requirements for path analysis, ensuring robust statistical evaluation of the relationships between Computer Vision Syndrome (CVS), visual fatigue, and sleep quality among nursing students.

### Data collection

The current study was based on data collected in January – May 2023 from undergraduate nursing students using a convenience sampling method. Only nursing students were invited to participate in the study, as they were considered a relevant group for investigating the relationships between computer vision syndrome, visual fatigue, and sleep disturbance, due to their extensive use of digital devices for academic purposes. By using convenience sampling, it was ensured that participants were readily accessible and could provide valuable insights into the impact of digital screen use on visual fatigue and sleep quality.

After ethical approval obtained from Nursing Research Ethics Committee of Faculty of Nursing at Damanhour University, Egypt, No (65-a) in 17/11/2022 the data collection process was carried out online through Google Forms, where participants completed self-administered questionnaires. The questionnaires included several scales designed to measure the variables of interest: Visual fatigue scale, sleep quality scale, computer vision syndrome tool. Nursing students voluntarily participated in this study and the online survey also guaranteed participants’ anonymity and confidentiality.

### Data collection tools

#### 1-Self-administered survey questionnaire

The self-administered questionnaire in this study gathers data on bio-socio-demographic information and health status, as well as specific behaviors related to video display terminal (VDT) usage. The socio-demographic section includes questions on age, gender and device usage. The health status section covers medication use, chronic diseases, and chronic eye conditions, such as conjunctivitis and glaucoma.

Additionally, the questionnaire collects detailed information about participants’ daily practices while using VDT devices like computers, laptops, and smartphones. This section includes questions on seating position, viewing distance, screen positioning, duration of use. The questionnaire also assesses awareness of computer vision syndrome, the use of eyeglasses or contact lenses. This structured approach aims to gather comprehensive data on the potential factors influencing CVS symptoms, visual fatigue, and sleep disturbances among nursing students.

#### 2-Computer vision syndrome questionnaire (CVS-S questionnaire)

To assess the occurrence of Computer Vision Syndrome (CVS) and its associated symptoms, a validated questionnaire (CVS-Q) was administered. This tool evaluates common ocular and visual discomforts, including eye strain, headaches, blurred or double vision, itching, dryness, excessive tearing, redness, pain, frequent blinking, foreign body sensation, burning or irritation, near-vision focusing difficulties, perceived vision deterioration, and light sensitivity. Participants rated symptom frequency using a three-point scale (“never,” “occasionally,” or “often/always”). Those reporting symptoms “occasionally” or “often/always” further rated intensity as “moderate” or “intense.” A composite score was derived by multiplying the frequency and intensity values for each symptom and summing the results. A total score of ≥ 6 indicated a positive CVS diagnosis [17].

#### 3-Sleep quality scale

The Sleep Quality Scale (SQS), a 28-item instrument, assesses six critical dimensions of sleep health: daytime dysfunction, post-sleep restoration, sleep onset and maintenance difficulties, morning alertness, and overall sleep satisfaction. Developed and validated by Yi et al., the SQS demonstrates strong psychometric properties, including high internal consistency (α = 0.92) and test-retest reliability (*r* = 0.81) [18]. Its results show significant convergence with the widely used Pittsburgh Sleep Quality Index, supporting its construct validity. Participants rate their sleep behaviors on a 4-point Likert scale (0 = “rarely,” 1 = “occasionally,” 2 = “frequently,” 3 = “consistently”). Notably, items within the restorative sleep and sleep satisfaction subscales are reverse scored prior to summation. Total scores range from 0 to 84, with elevated scores indicating more severe sleep disturbances [18].

#### 4-Visual fatigue questionnaire

The Visual Fatigue Questionnaire is a self-report tool that measures visual fatigue symptoms using a 10-point Likert scale. The questionnaire includes the following six questions, each question was answered on a seven-point Likert scale, with 1 indicating not at all and 10 indicating very serious. The sum of the scores of the six questions after finishing the experiment were used as the final subjective visual fatigue score [19].

All instruments used in this study the Computer Vision Syndrome Questionnaire (CVS-S), the Visual Fatigue Questionnaire, and the Sleep Quality Scale (SQS) were originally developed in English and underwent a structured process of cultural and linguistic adaptation for Arabic-speaking populations. This process followed established guidelines for cross-cultural instrument validation. Two bilingual experts independently translated each tool into Arabic, after which the translations were synthesized and reviewed by a multidisciplinary panel of five experts (including nursing and ophthalmology faculty) to ensure cultural relevance and conceptual equivalence. Content validity was formally assessed using the Content Validity Index (CVI). The scale-level CVI (S-CVI/Ave) was 0.92 for the CVS-S, 0.94 for the Visual Fatigue Questionnaire, and 0.91 for the SQS, indicating excellent content validity. A professional translator then performed a back-translation into English, and discrepancies were reconciled to maintain semantic accuracy.

To ensure clarity and comprehension, a pilot test was conducted with 21 undergraduate nursing students (excluded from the final sample), and no substantial revisions were needed. Reliability was assessed using Cronbach’s alpha, which demonstrated strong internal consistency: α = 0.91 for the CVS-S, α = 0.88 for the Visual Fatigue Questionnaire, and α = 0.89 for the SQS. Item-total correlations for all instruments exceeded 0.40. To further establish construct validity, inter-scale correlations were examined visual fatigue showed a strong positive correlation with computer vision syndrome (*r* = 0.675, *p* < 0.001), and both visual fatigue (*r* = 0.436, *p* < 0.001) and computer vision syndrome (*r* = 0.455, *p* < 0.001) were positively correlated with poorer sleep quality, supporting the convergent validity of the constructs.

## Data analysis

Data was analyzed using IBM SPSS version 29 and the PROCESS macro version 4.2 developed by Hayes (2018) [20]. Preliminary analyses included descriptive statistics (frequencies, percentages, means, and standard deviations) to summarize the study variables. Pearson’s correlation coefficients were computed to assess bivariate relationships among visual fatigue, Computer Vision Syndrome, and sleep quality. The reliability of all scales was confirmed using Cronbach’s alpha coefficients.

Mediation analysis was conducted using PROCESS macro (Model 4) with 5,000 bootstrap samples to test the indirect effect of visual fatigue on sleep quality through Computer Vision Syndrome as the mediator. This approach provides robust bias-corrected confidence intervals for indirect effects, following contemporary methodological standards. Unstandardized indirect effects, standard errors, and 95% confidence intervals were examined, with mediation considered significant if the confidence interval did not include zero. Statistical significance was set at *p* < 0.05 for all analyses.

Several procedural and statistical remedies were applied to ensure the quality of the data collected and mitigate the risk of CMB in the study results. Procedural remedies included reviewing the survey items with experts in the field and pre-testing them to ensure clarity while avoiding ambiguous or overly complex items. Clear instructions were provided to participants, emphasizing that there were no right or wrong answers. Participants were informed that the research was conducted solely for academic purposes, and their anonymity was guaranteed as no identifying information, such as names, was collected. Additionally, the order of survey items was randomized to minimize potential response biases [21].

## Results

Table 1 summarized socio-demographic and health-related factors among nursing students using visual display terminals (VDTs). The mean age was 19.96 years. Females comprised 67.3% of the sample, which aligned with general nursing program trends. Cell phones were the primary device (81.8%), followed by tablets (10.3%), personal computers (5.1%), and laptops (2.8%). Most students (95.3%) did not use medication, and 91.1% reported no chronic diseases, indicating a generally healthy population. Regarding eye health, 82.7% had no significant eye disorders, though 8.0% reported eye dryness, and smaller proportions reported myopia (1.4%), astigmatism (0.9%), and conjunctivitis (1.9%).

**Table 1:** Self-administered survey questionnaire (*n* = 214)

Self-administered survey questionnaire	n	%
**a) Socio-demographic information:**		
Age (Mean ± SD) 19.96 ± 1.43		
Gender		
Male	70	32.7
Female	144	67.3
Which type of device you are using		
Cell phone	175	81.8
Tablet	22	10.3
Personal computers	11	5.1
Laptop	6	2.8
**b) Health status:**		
Do you usually use any medication		
Yes	10	4.7
No	204	95.3
Do you have any chronic disease		
Yes	19	8.9
No	195	91.1
Do you have any chronic eye diseases		
Yes	37	17.3
No	177	82.7
What is your seating position while using the VDT devices		
My face is just at the level of the computer screen/other VDT devices	60	28.0
My face is not at the level of the computer screen/other VDT devices	154	72.0
What is the viewing distance while using the VDT devices		
Less than or equal to 50 cm	176	82.2
Greater than 50 cm	38	17.8
What is the level of the top of your computer screen/other VDT devices?		
Below the level of eyes	74	34.6
At the level of eyes	114	53.3
Above the level of eyes	26	12.1

Ergonomically, 72% of students reported their face was not at eye level with the screen, potentially contributing to poor posture and neck strain. Additionally, 82.2% sat ≤50 cm from the screen, a close viewing distance linked to increased CVS symptoms. While 53.3% positioned their screen at eye level (optimal), 34.6% had screens below eye level, and 12.1% above, both suboptimal for prolonged use. These findings highlighted practices that may have exacerbated visual strain and CVS symptoms.

Table 2 revealed that many nursing students had used computers or other VDTs for several years, with 42.5% reporting 5–10 years of use and 31.8% using them for ≥10 years. The mean duration of screen use was 6.74 years, indicating prolonged exposure that may have contributed to eye fatigue and CVS symptoms. Most students (72.9%) used devices daily (mean = 6.04 days/week), reflecting heavy reliance on digital tools for academic and personal tasks. Over half (52.3%) spent > 4 hours/day on devices (mean = 4.94 hours), increasing the risk of CVS symptoms such as eye strain and headaches. Despite this, only 18.2% were aware of CVS, while 81.8% lacked knowledge of the condition.

**Table 2:** Self-administered survey questionnaire (*n* = 214) “continues”

Self-administered survey questionnaire	n	%
Duration of computer use/other VDT devices (years)		
1 - < 5	55	25.7
5 - < 10	91	42.5
≥10	68	31.8
Mean ± SD 6.74 ± 2.86		
How many days per week do you use a computer/other VDT devices		
1	10	4.7
2	10	4.7
3	7	3.3
4	11	5.1
5	15	7.0
6	5	2.3
7	156	72.9
Mean ± SD 6.04 ± 1.80		
Approximately, how many hours per day do you work on the computer		
< 2	11	5.1
2–4	91	42.5
≥4	112	52.3
Mean ± SD 4.94 ± 1.92		
Are you aware of computer vision syndrome		
Yes	39	18.2
No	175	81.8
How often do you take a break while using a computer/other VDT devices		
Every 20 minutes of work	70	32.7
Every 60 minutes of work	72	33.6
Every 2 hours of work	35	16.4
More than every 2 hours	37	17.3
Do you use eyeglasses		
Yes	112	52.3
No	102	47.7
If yes What is the purpose of your eyeglasses (*n* = 112)		
For computer/other VDT device use	37	33.0
For vision	75	67.0
Do you use contact lens		
Yes	23	10.7
No	191	89.3
Do you use an anti-glare/VDT filter/blue light filter for your computer screen		
Yes	54	25.2
No	160	74.8

The data in table 3 showed that 50.9% of students have computer vision syndrome (CVS), with a total score of 6.56 ± 5.55, indicating moderate to severe symptoms. The remaining 49.1% of students reported no CVS (score < 6).

**Table 3:** Total score computer vision syndrome (*n* = 214)

Computer Vision Syndrome	n	%
Level		
No Computer Vision Syndrome ( < 6)	105	49.1
Have Computer Vision Syndrome (≥6)	109	50.9
Total score (Mean ± SD) 6.56 ± 5.55		

Table 4 data from the visual fatigue scale suggested that visual fatigue is a prevalent issue among participants, with a broad range of severity in terms of symptoms. The overall mean score of 18.06 **±** 11.89 indicated that visual fatigue is moderate.

**Table 4:** Visual fatigue scale (*n* = 214)

	Visual fatigue scale	Mean	SD
1	It is hard for me to see the screen clearly.	2.86	2.31
2	I have strange feelings in my eye	2.58	2.27
3	I have sore eyes such as tingling, irritability	2.59	2.32
4	The brightness of screen numbness my eyes	2.78	2.63
5	Looking at screen make me dizzy and fuzzy	2.76	2.53
6	I feel Headache	4.49	3.15
	Total score	18.06	11.89
	Mean score	33.6	15.2

Table 5 revealed that sleep-related headaches, concentration difficulties, and irritability were the most pronounced sleep issues among nursing students, indicating significant daytime impairment. In contrast, returning to sleep after awakening and appetite changes were less affected. The total score of 33.66 ± 15.2 suggested moderate overall sleep disturbances in this population.

**Table 5:** Sleep quality scale (*n* = 214)

Q	Sleep Quality Scale	Mean	± SD
Highest Impact Items			
1	Poor sleep gives me headaches	1.74 ± 1.08	
2	Poor sleep makes it hard to concentrate at work	1.50 ± 0.96	
3	Poor sleep makes me irritated	1.49 ± 1.09	
4	My fatigue is relieved after sleep	1.49 ± 0.96	
5	Poor sleep makes me easily tired at work	1.42 ± 0.99	
Lowest Impact Items			
1	I never go back to sleep after awakening	0.69 ± 0.90	
2	Poor sleep makes me lose my appetite	0.88 ± 0.98	
	Total score	33.66 ±15.26	

## Hypotheses testing

The results in Table 6 indicated significant positive correlations between the three variables. Computer Vision Syndrome (CVS) showed a strong positive relationship with the Visual Fatigue Scale (VFS) (*r* = 0.675, *p* < 0.001), meaning that increased severity of CVS symptoms was associated with higher levels of visual fatigue. Additionally, CVS was moderately correlated with the Sleep Quality Scale (*r* = 0.455, *p* < 0.001), suggesting that greater CVS severity was linked to poorer sleep quality. Similarly, visual fatigue was positively related to sleep quality (*r* = 0.436, *p* < 0.001), indicating that higher levels of visual fatigue were associated with worse sleep quality.

**Table 6:** Correlation between the studied variables (*n* = 214)

		Computer Vision Syndrome	Visual fatigue scale	Sleep Quality Scale
Computer Vision Syndrome	r			
	p			
Visual fatigue scale	r	0.675*		
	p	< 0.001*		
Sleep Quality Scale	r	0.455*	0.436*	
	p	< 0.001*	< 0.001*	

r: Pearson Correlationcoefficient.   *: Statistically significant at p ≤ 0.001

In Table 7 the regression model showed that both Computer Vision Syndrome and visual fatigue were significant predictors of sleep disturbances among nursing students. CVS had a stronger effect (β = 0.296, *p* = 0.001) than visual fatigue (β = 0.236, *p* = 0.004).

**Table 7:** Multivariate linear regression analysis for factors affecting sleep disturbance (*n* = 214)

Variable	B	β	t	p
Computer Vision Syndrome	0.816	0.296	3.638*	0.001*
Visual fatigue scale	0.302	0.236	2.891*	0.004*
R^2^ = 0.207, Adjusted R^2^ = 0.204, F = 32.8885^*^,*p* < 0.001^*^				

: Statistically significant at *p* ≤ 0.001

**Fig. 2:**
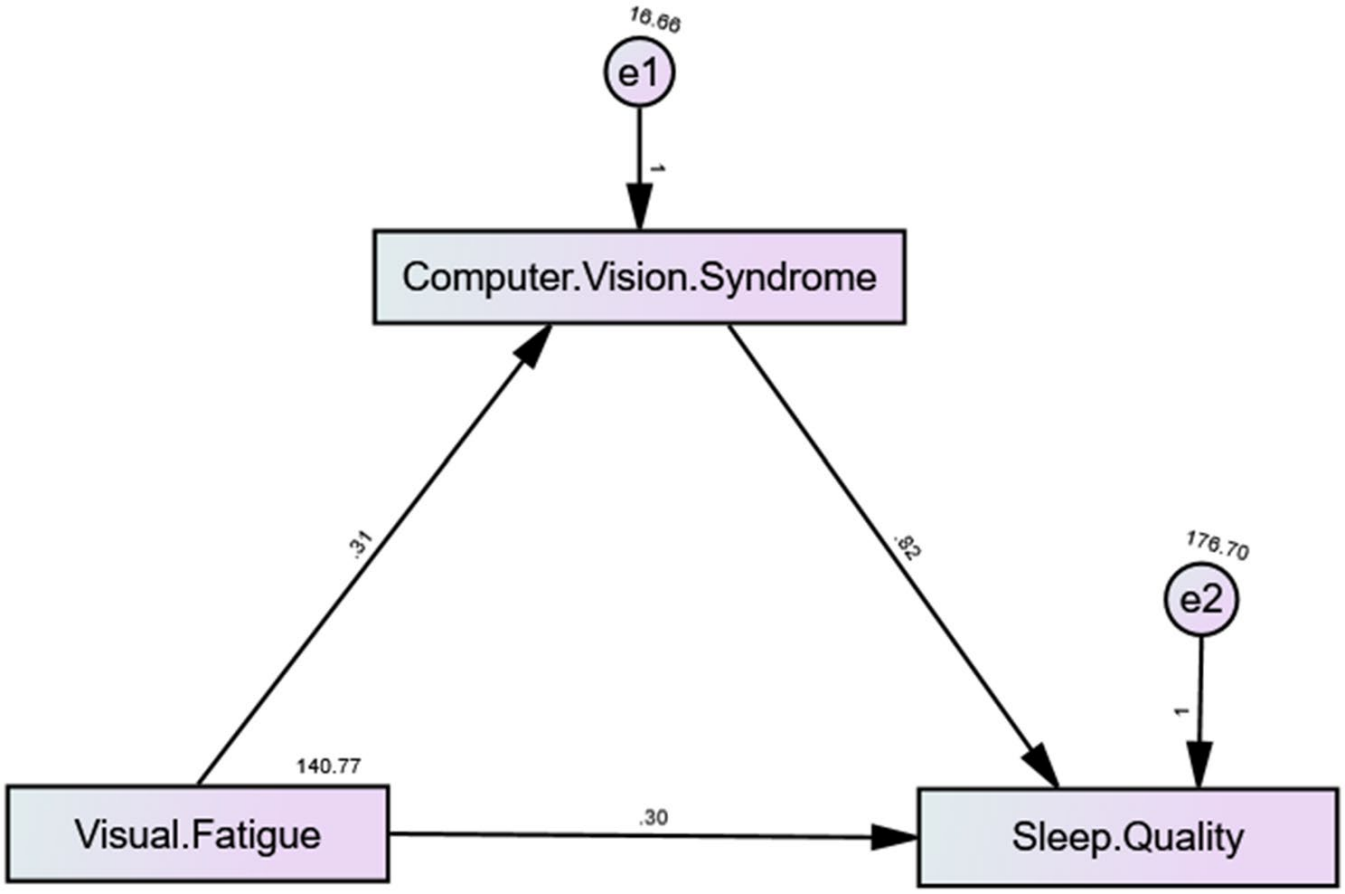
Path analysis Model with standardized path coefficients. * *p* < 0.05, ** *p* < 0.01, *** *p* < 0.001

The path analysis illustrated the relationships between computer vision syndrome, visual fatigue, and sleep quality. The path coefficient of 1.45 between CVS and Visual Fatigue indicated a strong positive relationship, suggesting that more severe CVS symptoms significantly increased Visual Fatigue. This, in turn, moderately influenced Sleep Quality, as shown by the path coefficient of 0.30. Additionally, CVS had a direct and substantial impact on Sleep Quality, with a path coefficient of 0.82, highlighting its significant role in sleep disturbances (Fig. 2).

Table 8 presented the results of the bootstrapped mediation analysis. All direct pathways were statistically significant. Visual fatigue demonstrated a strong, significant direct effect on CVS (B = 0.315, β = 0.675, *p* < 0.001). CVS, in turn, showed a significant direct effect on sleep quality (B = 0.816, β = 0.296, *p* < 0.001). Visual fatigue also had a significant direct effect on sleep quality (B = 0.302, β = 0.236, *p* = 0.004). Critically, the indirect effect of visual fatigue on sleep quality through CVS was statistically significant (B = 0.257, β = 0.200), as evidenced by a 95% bootstrap confidence interval that did not include zero [.131, 0.375]. This confirmed that CVS partially mediated the relationship. The total effect of visual fatigue on sleep quality was also significant (B = 0.559, β = 0.436, *p* < 0.001).

**Table 8:** Path analysis of the relationships among visual fatigue, computer vision syndrome, and sleep quality (direct and indirect effect

Path	Direct/Indirect	B	β	SE	t	p	95% CI Lower	95% CI Upper
Visual fatigue ➝ CVS	Direct	0.3148	0.675	0.0236	13.3230	0.000***	0.2682	0.3614
CVS Sleep ➝ Quality	Direct	0.8156	0.296	0.2242	3.6380	0.000**	0.3737	1.257
Visual fatigue ➝ Sleep quality	Direct	0.3023	0.236	0.1045	2.8914	0.004*	0.0962	0.5084
Indirect effect Visual fatigue to Sleep quality through CVS	Indirect	0.2568	0.200	0.0618	-	-	0.1312	0.3754
Total effect		0.5590	0.436	0.0793	7.0476	0.0000***	0.4027	0.715

Statical significance * p < 0.05, ** p < 0.01, *** p < 0.001

## Discussion

The purpose of this study was to explore the relationships between computer vision syndrome (CVS), visual fatigue, and sleep quality, and to test the mediating role of CVS between visual fatigue and sleep quality among nursing students. The results indicated moderate levels of all three variables. A significant positive correlation was found between CVS and visual fatigue, and visual fatigue was positively correlated with poorer sleep quality. Furthermore, path analysis confirmed that CVS statistically mediated the relationship between visual fatigue and sleep quality, suggesting that the severity of visual fatigue symptoms indirectly affects sleep quality through CVS.

Our findings align with established literature linking prolonged screen exposure to digital eye strain and its sequelae [11, 13]. The visual fatigue prevalent in digital learning environments stems from overstimulation under suboptimal ergonomic and lighting conditions, which can extend beyond ocular discomfort to impair cognitive processing and emotional regulation [22, 23]. Crucially, the physiological stress from this sustained near-work is a key mechanism, potentially contributing to heightened arousal and circadian rhythm dysregulation, thereby disrupting sleep [23].

While blue light exposure is a known suppressor of melatonin [24], our results indicate its sleep-disrupting effects are likely amplified by the presence of CVS and visual fatigue. These elements function interactively; visual fatigue may reflect and intensify the overall physiological burden of screen exposure, with CVS symptoms potentially sensitizing individuals to environmental light and thereby exacerbating sleep difficulties [25]. This suggests a cumulative model of risk beyond any single mechanism.

A vicious cycle appears to link CVS and poor sleep. Sleep deprivation can worsen CVS by reducing blink rates, increasing dry eyes and light sensitivity, and impairing ocular muscle recovery. Conversely, CVS-related discomfort and visual fatigue can disrupt circadian rhythms and delay sleep onset [26]. This bidirectional relationship creates a feedback loop that is particularly detrimental for students in digitally intensive programs.

A critical finding of this study is the profound knowledge gap regarding CVS, with 81.8% of nursing students unaware of the syndrome. This low digital health literacy is concerning, as it prevents students from connecting their symptoms to screen use and adopting protective behaviors. While prevalence rates vary across studies ‎ [27–29]‎, the persistence of this awareness gap points to a systemic failure in integrating digital wellness education into nursing curricula [28]. Addressing this is an urgent educational imperative; empowering future nurses with knowledge about CVS through targeted workshops, ergonomic training, and curriculum integration [29] is essential not only for their own well-being but also for their capacity to educate future patients in an increasingly digitalized healthcare landscape.

The high prevalence of CVS (50.9%) and visual fatigue (mean score 18.06 ± 11.89) in our sample confirms the significant burden of screen-related strain. The strong association and clinical overlap between CVS and visual fatigue suggest a cumulative strain on the visual system [30, 31] The variability in symptom severity underscores the influence of modifiable factors like ergonomics and individual susceptibility [32, 33], highlighting the need for both universal educational strategies and individualized interventions.

The reported mean sleep quality score (33.66 ± 15.26; 40.07% ± 18.17) indicates moderate disturbance, consistent with prior studies linking poor sleep to impaired academic performance and increased stress [34]. Importantly, this study supports a bidirectional relationship: sleep disturbances may both result from and exacerbate visual strain. CVS-related discomfort can impair sleep quality [17], while poor sleep can heighten fatigue, sensitivity to light, and visual discomfort creating a feedback loop particularly relevant to digitally active student populations [27].

The mediation analysis provides insight into the interconnected roles of CVS, visual fatigue, and sleep quality. CVS exerted both direct and indirect effects on sleep quality, with CVS emerging as a mediator. This finding aligns with prior research identifying CVS as a key pathway through which screen-related strain contributes to sleep disruption [35]. The indirect effect suggests that CVS does not only impair sleep through physiological mechanisms such as blue light exposure or melatonin suppression but also by amplifying visual fatigue, which itself is a known disruptor of circadian regulation.

The strong association between CVS and visual fatigue underscores their clinical and conceptual overlap, particularly among nursing students, who are frequently exposed to prolonged screen use during academic and clinical documentation tasks [11, 36]. The cumulative stress from sustained near-vision focus may exacerbate physical discomfort, increasing arousal and cognitive effort, thereby interfering with sleep onset and maintenance [37].

Lastly, current study confirms that Computer Vision Syndrome significantly mediates the relationship between visual fatigue and poor sleep quality in nursing students. These findings underscore the need for integrated digital wellness strategies in nursing curricula to protect student well-being and prepare a resilient future healthcare workforce.

## Limitations

This study has several limitations that should be acknowledged. The cross-sectional design limits the ability to establish causal relationships between Computer Vision Syndrome (CVS), visual fatigue, and sleep quality; therefore, longitudinal studies are recommended to clarify temporal dynamics and long-term effects. The focus on nursing students may restrict the generalizability of the results, and expanding samples to include other healthcare students or practicing professionals could provide a broader perspective. Although the instruments were rigorously translated and validated, they were not specifically designed for the nursing education context; thus, the development of population-specific tools would enhance contextual relevance. The use of convenience sampling may also limit generalization representativeness, highlighting the need for more robust sampling techniques such as stratified or random approaches in future research

Furthermore, there is the potential for self-selection bias, inherent in the convenience sampling method. Participants with heightened awareness of, or discomfort from, digital eye strain may have been more likely to enroll. This could lead to an overestimation of Computer Vision Syndrome prevalence and inflate the observed associations with visual fatigue and sleep disturbance. Finally, conducting the study in a single geographic region may restrict applicability to other cultural or institutional contexts, and multicenter or cross-national studies are recommended to improve external validity and broader relevance.

## Conclusion and recommendations

This study identifies a critical challenge in contemporary nursing education: the demonstrated pathway from digital eye strain to sleep disruption through Computer Vision Syndrome (CVS). The findings establish CVS not merely as a symptomatic complaint but as a significant mediator, revealing that prolonged screen exposure initiates a physiological process capable of compromising the restorative sleep essential for academic and clinical performance.

These results necessitate a shift from awareness to structured intervention. Integrating evidence-based digital wellness strategies blue light management directly into nursing curricula represents a fundamental step. Concurrently, institutional initiatives to optimize learning environments through glare reduction and the promotion of protective technologies are indicated.

Addressing this cascade is ultimately a question of professional sustainability. Treating CVS with formal recognition via early screening, practical education, and supportive policy can help preserve student well-being. A proposed action plan could solve the prevalence of CVS, integrate mandatory CVS screening and ergonomic education into the nursing program’s curriculum for early intervention. Also, providing students with practical digital wellness and sleep hygiene toolkits. In addition, advocate for institutional policies that mandate screen-free breaks and blue-light filtering software. This cohesive strategy of screening, education, and policy will directly mitigate the health impacts of digital learning and protect our future nursing workforce.

## Data Availability

The datasets used and/or analyzed during the current study are available from the corresponding author on reasonable request.

## Abbreviations

CVS: Computer Vision Syndrome
STROBE: Strengthening the Reporting of Observational Studies in Epidemiology
VDT: video display terminal
SQS: Sleep Quality Scale
CVS-Q: Computer vision syndrome scale
CMB: Common method bias
